# Comparing apples with apples: A proposed taxonomy for “Community Health Workers” and other front-line health workers for international comparisons

**DOI:** 10.1371/journal.pgph.0004156

**Published:** 2025-02-06

**Authors:** Stephen Hodgins, Uta Lehmann, Henry Perry, Nicholas Leydon, Kerry Scott, Smisha Agarwal, Hannah Marcus, Rajani Ved, Elijah Olivas, Madeleine Ballard, Dickson Mbewe, Margaret Odera, Sherlie Petit Homme, Benard Otieno, Pasipano Wutete, Angeline Chikumba, Prossy Muyingo, John Kyakuha, Emmanuel Harcourt, Morseda Chowdhury, David Musoke, Thadee Niyoyitungira, Abimbola Olaniran, John Koku-Awoonor Williams, Lívia Milena Barbosa de Deus e Méllo, Romário Correia dos Santos, Isabela Cardoso de Matos Pinto, Ram Shrestha, Salim Sadruddin, Melanie Morrow, Eric Sarriot, Maryse Kok, Bhanu Pratap

**Affiliations:** 1 School of Public Health, University of Alberta, Edmonton, Alberta, Canada; 2 School of Public Health, University of the Western Cape, Cape Town, South Africa; 3 Bloomberg School of Public Health, Johns Hopkins University, Durham, North Carolina, United States of America; 4 Bill & Melinda Gates Foundation, Seattle, Washington, United States of America; 5 Bloomberg School of Public Health, Johns Hopkins University, Toronto, Ontario, Canada; 6 Bloomberg School of Public Health, Johns Hopkins University, Baltimore, Maryland, United States of America; 7 Consilient Research, Nairobi, Kenya; 8 Bill & Melinda Gates Foundation, Delhi, India; 9 Community Health Impact Coalition, London, United Kingdom; 10 Community Health Worker, Ministry of Health, Kasungu, Malawi; 11 Community Health Worker, Ministry of Health, Nairobi, Kenya; 12 Community Health Worker, Ministry of Health, Port-au-Prince, Haiti; 13 Community Health Worker, Ministry of Health, Migori, Kenya; 14 Community Health Worker, Ministry of Health, Gutu, Zimbabwe; 15 Community Health Worker, Ministry of Health, Kutama, Zimbabwe; 16 Living Goods Community Health Worker, Mityana, Uganda; 17 Community Health Worker, Ministry of Health, Jinja, Uganda; 18 Mailman School of Public Health, Columbia University, New York City, New York, United States of America; 19 BRAC, Dhaka, Bangladesh; 20 Makerere University, Kampala, Uganda; 21 Africa CDC, Addis Ababa, Ethiopia; 22 London School of Hygiene & Tropical Medicine, London, United Kingdom; 23 Formerly of Health Service, Accra, Ghana; 24 Ministry of Health, Brazilia, Brazil; 25 College of Applied Food and Dairy Technology, Kathmandu, Nepal; 26 Formerly World Health Organization, Geneva, Switzerland; 27 The Health Systems Strengthening Accelerator/ICF, Rockville, Maryland, United States of America; 28 Agence Régionale de Santé Nouvelle-Aquitaine, Bordeaux, France; 29 Liverpool School of Tropical Medicine, Liverpool, United Kingdom; 30 IFRC, Geneva, Switzerland; Faculty of Medicine, Universitas Sebelas Maret, INDONESIA

## Abstract

This paper proposes a taxonomy for Community Health Workers (CHWs) and others engaged in front-line community health activities, encompassing formally-employed workers extending government primary health care (PHC) service delivery as well as a range of other actors with roles at the nexus of government PHC and communities. The taxonomy is grounded in current definitions from the World Health Organization and the International Labor Organization, and proposes some refinements for future iterations of guidance from these agencies. The designation, “Community Health Worker” is currently used to cover a broad range of roles. Furthermore, there are programs engaging workers or community members in roles closely adjacent to those generally recognized as CHWs that use other designations, not commonly included under the rubric of “CHW”. This potentially confusing range of roles and nomenclature leads at times to over-generalizations, applying insights and principles relevant for one type of worker or community member that are not necessarily relevant for another. It also leads to a failure to consider occupational groups not commonly thought of as CHWs—but engaged in PHC service delivery at the most peripheral level—in community-based-PHC planning and management arrangements. Building on ILO and WHO classifications and standards, a further clarification of terms and a taxonomy is proposed, with the intention of contributing to clearer communication and shared understanding and, ultimately, sounder community health policy, program planning, and implementation; and more substantial progress towards Universal Health Coverage.

## Introduction

Mechanisms that enable primary health care (PHC) to effectively reach and engage with communities can have important benefits for population health [1]. In many settings—primarily in low and lower middle-income countries (LMICs) but also in many higher income countries—“community health workers” (CHW) have played a central role in this effort. The CHW concept has been around for many decades. They are known by many names and have played a wide range of roles. Over the past 10–15 years there has been a resurgence of interest in their potential contribution, most recently during the acute COVID response (with new duties assigned to them), and this has been accompanied by calls for greater formalization of their roles and for better support and working conditions [2,3].

With increasing attention to the potential contribution of CHWs and increasing numbers of countries seeking to incorporate CHW cadres within the PHC workforce, one of the challenges in the discourse and policy debates around CHWs is that there are very different understandings on the definition of CHWs. Approximately 45 different descriptors have been used to describe CHW cadres around the world, but they remain conceptually poorly defined [4]. As pointed out in the 2018 CHW guidance from WHO [5],

“Unclear nomenclature and classification complicate the policy discourse on CHWs: the term “community health workers” is often used in a non-specific way, referring to a diverse typology of lay and educated, formal and informal, paid and unpaid health workers.”

Given that CHW programs have emerged largely in response to local needs, and these needs and hence roles vary and have changed over time, this diversity in how CHWs are defined and classified is understandable. Authors have constructed CHW taxonomies based on several considerations:

- the roles CHWs may play, specifically in immunization programs [6],
- education level required and duration of pre-service training [7,8],
- relative emphasis on promotive vs. preventive vs. curative tasks [8], and
- relative strength of provisions for supervision and other health system support functions [8].

These proposed taxonomies draw attention to important differentiating characteristics of CHW programs; however, we believe there is still imprecision in where we draw the boundaries around those we are calling CHWs and how we differentiate across different types, under the broad heading of “CHW”. With further clarification of terminology and taxonomy, expected benefits include:

- clearer communication [9,10],
- more standardized reporting [11–14],
- clearer framing for synthesizing evidence from program experience [12,15], and
- better transferability of lessons learned across different settings and types of programs.

There are also other occupational groups whose work aligns closely with what is understood to be the role of a CHW (and how this role is described by the International Labor Organization (ILO), as discussed below) but who are not typically considered CHWs. For example, in Uganda, there are “*nursing assistants*”, “*enrolled nurses*”, and “*enrolled midwives*” staffing “Health Center IIs” who play a role quite similar to that of *Health Extension Workers* associated with “health posts” in Ethiopia, who are widely seen as CHWs. However, because these Ugandan cadres are not normally thought of as CHWs, they were passed over when the country began to roll out “integrated community case-management” of childhood illness (iCCM), in contrast to implementation of iCCM in Ethiopia, where this was done primarily through *Health Extension Workers*.

And then there is the “worker” portion of this label. For paid full-time or regular part-time staff, “worker” seems an appropriate term. But there are diverse arrangements for how PHC engages with members of the community. Often there are community members who are regularly or intermittently engaged in some way with government PHC services and this may or may not include a service delivery role. In some programs, such individuals may receive honoraria, stipends or other monetary incentives. Does that make them “workers”? As we will discuss below, use of the term “volunteer” as blanket term to describe those who are not formally employed may also be problematic.

Within actual country programs, it may not matter if, for a particular role, a different job title is used than that used in another jurisdiction. However, if we want to draw potentially transferable lessons from one country’s experience to apply in another or if we are engaged in developing global or regional program guidance, we need to be comparing apples with apples, and oranges with oranges.

One important factor adding complexity to efforts to develop definitions and terminology for use across diverse settings is the diverse range of formally-employed (usually full-time) workers and various less formally-engaged, less-than-full-time community actors who are connected in some way with government PHC. In this paper, we present a taxonomy that segments across a small number of dimensions, the most important of which is the distinction between—on the one hand—formally-employed workers and—on the other—community members who are actively engaged with government PHC on at least an intermittent basis, but who are not formally employed (we acknowledge the important role, in some settings, of NGO-associated CHWs; in future work, it may be worthwhile to better characterize a taxonomy of such programs. However this paper largely focuses on large-scale government programs).

## Methodologic note

This review can be understood as the synthesis of a consensus process. It is not intended as a research article or systematic review. The process in developing the paper includes efforts stretching back several years. This has entailed the development, by various of the co-authors of this paper, of a set of comprehensive program case studies of CHW cadres from 29 countries [16]. It also included work done in 2021, commissioned by the Bill & Melinda Gates Fund, to derive a CHW “typology”, based on information on large-scale government CHW programs and a modified Delphi process seeking perspectives from a diverse range of global and country-level CHW program stakeholders. This process continued in 2023 with multiple rounds of consultation, including a Delphi-type survey, webinars, and an iterative process of circulating drafts to stakeholders, seeking their perspectives, and revising accordingly. This is reflected in our author list, which includes 33 individuals from 19 countries.

## Community-based primary health care

In this paper, we are concerned with “community-based primary health care” (CB-PHC). We recognize, however, that there are categories of workers otherwise meeting definitions for CHW whose work is not community-based, e.g., peer counsellors working with people living with HIV but based in clinics and hospitals rather than the community. Furthermore, in the future we can expect development of PHC services, in which our definitions of “community” will not be restricted to physical localities such as villages or neighborhoods; “community” may be understood to include groups of people not necessarily living close together but sharing other characteristics.

For purposes of this article, we are using the term CB-PHC mainly to refer to public-sector PHC services provided below the level of health center; we are *not* looking at services at the level of “health centers” or “primary health care centers”. In this paper we restrict our use of the term “health center” to health facilities with at least some staffing by professional-level health workers (as defined by ILO) and normally serving populations of 20,000 or more, recognizing that in some countries (e.g., Uganda) the term, “health center”, is also used at a more peripheral level, more or less equivalent to Ethiopia’s Health Posts.

Service delivery modalities or platforms at the CB-PHC level—as we are using this term—may include health posts or dispensaries, outreach/mobile and home-based services. There may be some professional-level providers working at this level, particularly in higher-income countries, but typically services are delivered primarily by “associate professional” workers (using the classification from the ILO [17], discussed in more detail, below). In many countries, this is an important component of rural government PHC and is commonly organized in service delivery units covering populations of about 3,000 to 10,000. Where such a service delivery level exists in urban settings, the populations covered may be larger. Furthermore, we recognize that the way we are defining CB-PHC for the purposes of this paper offers a rather narrow perspective, focusing largely on provision of PHC services at the most peripheral level of government health services. Other entirely legitimate definitions of CB-PHC [18] focus attention less exclusively on services provided by the government and more on actions by members of the community.

## Definitions for CHWs

We will begin by briefly reviewing recent definitions from the World Health Organization (WHO) and ILO (see Table 1, below). In the following sections, for the portion of the CHW spectrum comprising formally employed workers, we will draw particularly on classifications and definitions from the ILO’s International Standard Classification of Occupations: ISCO-08 [17]. We believe that the WHO and ILO definitions serve as a good starting point for characterizing CHW programs, although further clarification and elaboration is needed.

**Table 1:** Comparison of “Community Health Worker” definitions.

	Education & training	Place of residence/identity	Remuneration	Services provided
ISCO-08: 3253 [17]	As “associate professional”, would generally require secondary school graduation & usually ≥1 year of training.	Not mentioned	Paid (as for all occupations listed in ISCO-08)	Provides health education, referrals, support, preventive care and help in accessing curative care & social services
Scott [19]	Not professionals; <2 years training but at least some training	Based in communities, “conducting outreach from their homes & beyond PHC facilities or based at health posts not staffed by doctor or nurse”	Paid or volunteer	Not mentioned
WHO [5]	Reiterates ILO ISCO-08 & Scott. Not professionals; <2 years training but at least some training; lower levels of formal education & training than professional health care workers such as nurses & doctors.	Member of the local community	Paid or volunteer. “Financial package commensurate with job demands, complexity, number of hours, training & roles they undertake.”	Prevention, diagnosis, treatment & care, e.g., for HIV, TB, malaria, other communicable & non-communicable diseases; reproductive, maternal, newborn & child health services; hygiene & sanitation; ensuring client adherence to treatment; rehabilitation & services for people affected by disabilities; advocating & facilitating underserved groups’ access to services

To be clear, we are not making the case for standardizing CHW *programs*; instead, we believe that some further standardization of *terminology* can be helpful for drawing potentially transferable lessons between programs (positive and negative) and for informing development of global strategy and program guidance. Standardization of terminology may also help national governments that are establishing or institutionalizing CHW programs to clarify scopes and roles within CB-PHC. We are concerned, here, with the language used for evidence synthesis, for global guidance, and perhaps most importantly for exchanges of information and insights between countries. Within a country, however, there may be very good reasons to continue to employ the designations and categories currently in use.

The ILO, in ISCO-08 [17], classifies “community health workers” (3253) at the level of *associate professional/ technician*, and specifies that CHWs:

“provide health education and referrals for a wide range of services, and provide support and assistance to communities, families and individuals with preventive health measures and gaining access to appropriate curative health and social services. They create a bridge between providers of health, social and community services and communities that may have difficulty in accessing these services”.

Scott et al. [19], in a review conducted for WHO, define CHWs as:

“health workers based in communities (i.e., conducting outreach from their homes and beyond primary health care facilities or based at peripheral health posts that are not staffed by doctors or nurse), who are either paid or volunteer, who are not professionals, and who have fewer than 2 years training but at least some training, if only for a few hours.”

This definition is used in the 2018 WHO guidance [5] for which the review by Scott et al. was commissioned. As this definition comprises “volunteers” as well as formally-employed, paid CHWs, it is broader in scope than the ISCO-08 definition (Table 1).

Largely in line with the definition from Scott and the WHO, we will now offer a synthesis which includes both formally-employed (generally full-time) staff as well as others not formally employed who have commonly been referred to in the literature as “volunteers”, although this term (normally understood as meaning “someone who does work without being paid for it”) can be misleading given the variety, across programs, in time commitment expected and remuneration provided, and in the extent to which the role is primarily defined in relation to the community vs. the health system. Communities may bring significant material support to CHWs (paid or unpaid) who they see as bringing value to the community, in the same way those communities might support others who contribute to the community, such as teachers or religious leaders.

For formally employed workers, we are proposing adherence to the schema proposed by ISCO-08, including the principle of classification based primarily on the tasks involved rather than the credential or duration of training required. As discussed below, we also note that in some programs, CHWs who are involved in service delivery on a regular basis, but not formally employed, may have tasks otherwise corresponding with the ISCO-08 CHW designation.

Details in this paper on specific cadres are drawn mainly from a set of country program case studies by Perry et al. [16], supplemented by further documentation on these programs done by several of the authors in the context of a contract with the Bill and Melinda Gates Foundation, on CB-PHC.

## Differentiating across CHW programs

### Formally-employed workers in CB-PHC

Terms of service for formally-employed CB-PHC staff may be *permanent*—typically with benefits such as pension and health insurance—or *time-bound*, e.g., an annual contract, potentially renewable, generally with limited or no supplementary benefits. The hiring authority is commonly a national or sub-national civil service or local government. In some settings, non-governmental organizations (NGO) engage CHWs under service agreements with public sector agencies, with various arrangements for remuneration.

Low health professional density is common in LMICs, particularly in rural areas. In the Africa region, for example, there are 2·9 medical doctors and 12·9 nursing and midwifery personnel per 10,000 population (ratios would be considerably lower in rural areas); in the Europe region, the corresponding figures are 36·6 and 83·4 (WHO Global Health Observatory [20]). A common strategy to address this gap has been delegating tasks usually performed by health professionals to lower-level occupational groups. Indeed, such “task shifting” or “task sharing” has also been effectively deployed for improving service access in some settings in high-income countries, e.g., engaging physician assistants and nurse-practitioners. There is some evidence that this modality of service provision has benefits even when there is no shortage of clinicians, for example because of improved access, reduced opportunity costs, and greater cultural and linguistic appropriateness.

Many workers engaged in service delivery in CB-PHC fall under what ILO categorizes—in ISCO-08—as the *associate professional/technician* level (3000). Under ISCO-08, at this level the entry requirement is typically secondary school graduation and the duration of pre-service training is usually at least a year. However, as explained below (Table 2), categorization under ISCO-08 is primarily based on the content of the work rather than on the credential or duration of training.

**Table 2:** ISCO “skill levels” and designation as “professional” vs. “associate professional”.

ILO defines “skill level” as “a function of the complexity and range of tasks and duties to be performed” **Skill level 1** – simple routine physical or manual tasks. For some occupations, completion of primary education may be required. **Skill level 2** – operating machinery, equipment; maintenance and repair; ordering and storage of information. Examples: butchers, bus drivers, secretaries, accounts clerks, and sewing machinists. Education typically required – first stage of secondary. On-the-job and/or specialized vocational training. **Skill level 3** (Associate professional/ technician) – complex technical and practical tasks, e.g., ensuring compliance with health and safety regulations, preparing estimates of quantities and costs for specific projects, performing technical functions in support of professionals. Secondary school graduation. Typically 1–3 years of post-secondary education at a higher educational institution (in some cases relevant work experience and on-the-job training may a substitute for formal education). Examples: shop managers, lab technicians, legal secretaries, radiographers, and computer technicians. **Skill level 4** (Professional) – tasks requiring complex problem-solving, decision-making, creativity, based on extensive theoretical and factual knowledge. Typically 3–6 years of post-secondary education. Examples: civil engineers, computer systems analysts, school teachers, medical practitioners, and nurses.

In this paper, we largely restrict our use of the term “professional” to its meaning as used in ISCO-08 (see Table 3), rather than how the terms “professional” and “professionalized” have been used in recent global discourse on CHW programs, referring to salaries, supervision, accreditation, etc.

**Table 3:** Task descriptions of relevant “associate professional” occupational groups from ISCO-08 [17].

**3253 – Community health workers**…provide health education, referral & follow-up, case management, basic preventive healthcare & home visiting services to specific communities. Provide support & assistance to individuals & families in navigating health & social services system. Tasks can include: a) providing information to families & communities on … health issues including nutrition, hygiene, infant & child care, immunizations, family planning, risk factors & prevention of common infectious diseases, poisoning, first aid for treatment of simple & common ailments, substance abuse, domestic violence & other topics; b) visiting families in their home to provide information on available health, social & other services & support them in gaining access to these services; c) visiting families who do not usually access medical establishments to regularly monitor conditions such as pregnancy, child growth & development, & environmental sanitation; d) distributing to households supplies for the prevention & treatment of endemic diseases such a malaria, pneumonia & diarrheal diseases, & instructing family & community member in the use of these products; e) conducting outreach to groups not usually accessing medical establishments with information & basic medical supplies for prevention & management of certain health conditions for which they are most at risk, such as HIV/AIDS & other communicable diseases; & f) collecting data from household & communities not usually accessing medical establishments for purposes of patient monitoring & referral & reporting to meet health regulations.	**3221 – Nursing associate professionals**…provide basic nursing & personal care for people in need of such care due to aging, illness, injury or other physical or mental impairment. Generally work under the supervision of & in support of medical, nursing & other health professionals, implementing health care, treatment & referral. Tasks can include: a) providing nursing & personal care & treatment, & health advice to patients according to care plans established by health professionals; b) administering medications & other treatments to patients, monitoring patients’ condition & responses to treatment, & referring patients & their families to health professional for specialized care as needed; c) cleaning wounds & applying surgical dressings; d) updating information on patients’ condition & treatments received in record-keeping systems; e) assisting in planning & managing the care of individual patients; & f) assisting in giving first-aid treatment in emergencies.	**3222 – Midwifery associate professionals**…provide basic healthcare & advice before, during & after pregnancy & childbirth. They implement care, treatment & referral plans usually established by medical, midwifery & other professionals. Tasks can include: a) *providing advice* to women, families & communities on health, nutrition, hygiene, exercise, birth & emergency plans, breast-feeding, infant care, family planning & contraception, lifestyle & other topics related to pregnancy and childbirth; b) assessing progress during pregnancy and child-birth, & recognizing signs and symptoms requiring referral to a health professional; c) providing delivery care, usually only in the absence of identified potential complications or assisting medical doctors or midwifery professionals with delivery care; & d) providing care & support to women & newborns following childbirth, monitoring health status & identifying signs requiring referral to a health professional

CHWs in many programs do not neatly fit within any one of the 4 skill levels in the ILO scheme, outlined above. Some of their tasks are of a complexity consistent with skill level 3. However, typically the level of education and duration of preservice training required are lower than what is common for level 3. ILO specifies, however, that “formal education and training requirements are only one element used for determining skill level” and, further, that for some occupations, formal education and training requirements differ between countries. Specifically, they note this is the case for primary school teachers and nurses; in some countries, university degrees are required for these occupations, in others, they are not. If categorization by level were based primarily on training requirements, this could result in school teachers and nurses in some countries being classified under the “professional” category and, in others, under “associate professional”. In the interest of improving international comparability, ILO has resolved this inconsistency by ruling that, in assigning formal skill level, job content (tasks and duties) be given priority over education and training requirements. As such, school teachers and registered nurses are classified under “professional,” rather than “associate professional,” even for countries where university-level credentials are not required for these occupations. By this logic, CHWs whose tasks align well with those specified by ILO for 3253 (see Table 3) would be classified under skill level 3, i.e., “associate professional”, even in programs where the required level of education and training may be lower than typical for this level. In its current work revising ISCO, ILO may want to consider recent evidence suggesting that training and experience, rather than formal education, are better predictors of CHW performance [21].

ISCO-08 also has a lower skill-level occupational group (at level 2) with functions somewhat analogous to those of CHWs: “home-based personal care workers” (5322), defined as: “workers who provide personal care [such as assistance with feeding, bathing, grooming, personal hygiene, transfers, e.g., from bed to walker] to residents of independent living units, generally without permanent medical or nursing supervision.” In future revisions to ISCO, potentially the task description for 5322 could be expanded such that his category could be used for some CHW cadres.

In some country programs there are workers engaged in governmental PHC below the health center level (i.e., CB-PHC) who would be categorized under ISCO-08 as *professional*, not associate professional. Indeed, over time—with rising educational levels and associated “credential creep”—in many countries there has been a shift to an increasing number of professional staff at this level (see Table 4). Examples of professional level staff working in CB-PHC include community-based professional nurses (ISCO-08 code: 2221) and midwives (2222), whose work is PHC/prevention-focused (although it may also involve curative services), serving as public health nurses. In some country programs there are also health professionals at CB-PHC level who can be classified as “paramedical practitioners” (2240), such as *Clinical Officers* in some African countries (e.g., Zambia, Malawi, Mozambique), *Health Assistants* in Nepal, and *Sub-Assistant Community Medical Officers* in Bangladesh. However, in most countries those with such designations are more commonly deployed at health center or hospital levels. These occupational groups, like professional nurses, generally have at least 3 years of pre-service training. Nigeria’s Community Health Extension Workers (CHEWs)—often considered a type of CHW—could also be classified as professionals, falling in the category of “paramedical practitioners” (ISCO-08:2240), given their 3-year duration of pre-service training and a scope of practice spanning that of CHW and paramedical worker. Similarly, WHO [22] has identified Mid-Level Health Workers, a category in which Nigeria’s CHEWs would fit well. Note that we are not proposing use of the “CHW” designation for these ISCO-08 2000-level “professional” workers (though we would include them under a broader designation such as “Frontline PHC Workers”).

**Table 4:** “Task-shifting” and “credential creep”.

Both of these terms concern credentials and scope of practice. In some sense, they are the inverse of each other. Under task-shifting (or “task-sharing”) arrangements, health workers take on tasks more typically performed by those with longer training and more advanced credentials. By contrast, with credential creep, over time a professional role or scope of practice formerly requiring a shorter duration of training and a lower-level credential requires an increasingly longer duration of training and higher-level credentials. Both can be seen as a response to availability of suitably qualified workers. In circumstances of low density of health workers able to perform certain tasks, task-shifting to workers with lower-level credentials may render such services more readily available, e.g., in settings with very low density of obstetricians, given suitable training a general practitioner’s scope of practice may be expanded to include caesarean deliveries. In settings where the level of schooling has substantially increased and more young adults are pursuing post-secondary education, higher-level credentials may become the new standard for certain professional roles, e.g., in settings where, formerly, registered nurses trained for 3 years in a nursing school, they may now be required to do 4-year university degrees. In many low- and lower middle-income countries, the density of more highly credentialed, specialized health workers is low, especially in rural areas. Under such conditions, task-shifting can be an effective strategy for making services more widely available, provided that there is commensurate training and support. Similarly, in higher-income countries, such a strategy may also be appropriate, notably in sparsely populated remote areas. In almost all countries, there have been progressive improvements in the proportion of young people completing schooling and going on to post-secondary training. With such changes, the pool of more highly qualified graduates of health worker training programs grows and higher credentials may become standard for roles previously requiring shorter training and lower-level credentials.

The associate professional/ technician occupational level is where ISCO-08 places “community health workers” (ISCO-08 code: 3253). There are other ISCO-08 occupational categories which, in principle, could be applied to at least some of those working in CB-PHC, notably “nursing associate professionals” (3221) and “midwifery associate professionals” (3222). Indeed, in many countries there are categories of workers with titles such as *enrolled*, *auxiliary* or *assistant nurse* or *midwife* working in CB-PHC (most with 2 academic years of pre-service training). The tasks listed in Table 3, for these two occupational categories, are most closely aligned with the role of such workers based in hospitals or health centers and working under the direct supervision of professional nurses or other health professionals. However, commonly their counterparts working in CB-PHC have tasks more closely approximating those of the ISCO-08 category “community health worker” (3253), as outlined in Table 3, albeit with more task-shifted clinical functions than we see in this outline of CHW tasks. The actual scope of practice of auxiliary-nurse-type workers (3221, 3222) engaged at health post level and below can be considered to fall in an overlap area spanning 3253 and 3221/3222. However, currently, for none of these 3 categories do the ISCO-08 task descriptions fully reflect the actual scope of practice and level of autonomy of most such workers.

Examples of such occupational groups with roles in this overlap area include *Public Health Midwives* in Sri Lanka, *Auxiliary Nurse-Midwives* (ANMs) in India based in Health and Wellness Centers, ANMs in Nepal based in Health Posts, and *Enrolled Nurses* working at CB-PHC level in many African countries. Conceptually (and for planning purposes), we propose that it is appropriate to consider such cadres together with others more conventionally classified as CHWs. However, how the various occupational groups are designated will be determined by history and other contextual factors; we are not trying to prescribe how they should be officially labeled at this level, within a country.

Pre-service training for formally-employed CHWs and other front-line associate professional health workers with similar scopes of work typically ranges from a few months up to about 2 academic years, in many cases in some kind of a nursing or technical college or training institution. Training may be done before hire (as it is for *ANMs* in India and Nepal) or after hire (e.g., for *Health Extension Workers* in Ethiopia and for *Agentes Comunitários de Saúde* in Brazil). Given their work responsibilities, based on ISCO-08, it can be argued that even those workers in programs in which entry may not require having completed secondary education and pre-service training may be as little as 2 or 3 months (i.e., substantially shorter than what is typical for the ISCO-08 associate professional/ technician category) would still appropriately be designated as “associate-professional”.

### Other community members actively engaged with governmental PHC but not formally employed

This group comprises actors with a diverse range of engagement, beyond a formal employer-employee relationship. The major categories we have identified include:

**Governance or community action roles** (but not a service delivery role *per se*): e.g., as members of village health or development committees, health facility committees, locally-elected bodies; and self-help, community action, or mothers groups. Rather than extension workers providing services *to* the community, many falling into this category can best be understood as people *from* and *acting on behalf of* the community. Usually there is no remuneration for such roles, although some may offer livelihood opportunities (e.g., micro-credit loans) and in some settings they are supported in other ways by their communities. Depending on the local program context and history, some are labeled CHWs; many are not.

**Episodic, occasional or time-limited involvement in service delivery:** e.g., helping mobilize community members for monthly, semi-annual or annual outreach health events. Their role may include dispensing health commodities (e.g., vitamin A to infants and young children, insecticide-treated mosquito nets, presumptive ivermectin treatment for onchocerciasis, deworming medications, oral polio vaccines). In some settings, special cadres of CHWs have been recruited, trained and deployed on a time-limited basis to undertake epidemic-control-related tasks. In other settings, community workers are recruited and deployed on a seasonal basis for vector-control activities, notably indoor residual spraying. Some NGO CHWs functioning largely independently of government CB-PHC nevertheless closely coordinate with government PHC for specific activities, e.g., supporting periodic campaign-type outreach (e.g., support provided by BRAC’s Shasthya Shebika program in Bangladesh for immunization campaigns). There are diverse arrangements for remuneration.

**Regular service delivery role:** this can entail a significant number of hours every week. Again, there are variable arrangements for remuneration, including daily wages, honoraria, stipends, allowances (e.g., for transport, meals, training), sales commissions (in BRAC’s NGO model), and performance-based incentives, e.g., for accompanying a woman to hospital to give birth or when a tuberculosis patient they have been supporting completes treatment. In some programs, involvement can amount to 10+ hours every week; we are proposing, as elaborated in the next section, that CHWs with this level of involvement be classified as “regular service-delivery CHWs, not-formally-employed” (RSD-CHW). Commonly, at this level, initial training can last up to several weeks. In programs with partner support, there is often provision for regular in-service training. In India, ASHAs fall in this category; they generally have fairly complicated mixed remuneration, partly performance-based (and this varies between states), but they are not salaried or formally employed. Pakistan’s Lady Health Workers used to have a similar status (although remuneration was not primarily performance-based) but they are now recognized as formally-employed civil servants. There can be a spectrum of time commitment involved, with some programs having many CHWs falling between what we have labeled episodic and “regular”, i.e., engaged in such service more often than monthly, but generally putting in fewer than 10 hours a week and having discretion over when they engage and how much time they commit. Rwanda’s *Polyvalent CHWs* and Nepal’s *Female Community Health Volunteers* fall in this **intermediate** category. Many, though certainly not all, programs having CHWs in this intermediate range (i.e., engaged in at least some PHC-related activities most weeks, but generally less than 10 hours/week) have significant support from an externally-funded partner agency.

## A proposed taxonomy

**Fig 1:**
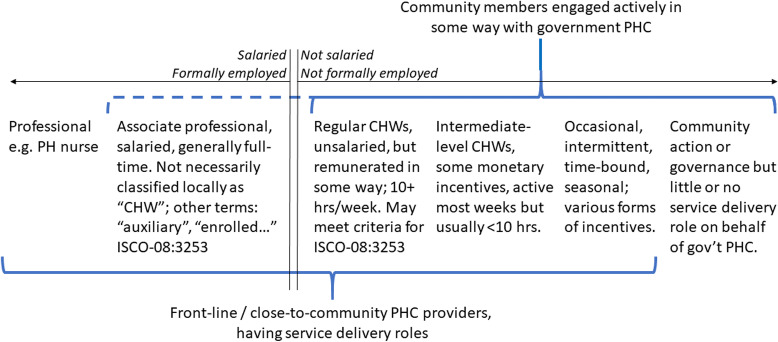
Spectrum of PHC players.

The full spectrum of health workers and community members involved with CB-PHC is reflected below in Fig 1. All of these players, where present, need to be adequately taken into account in government human resource planning and management for PHC, including program monitoring, evaluation, and formal reporting.

The dotted section of the line is intended to suggest that, depending on the setting, salaried, associate-professional-level workers are sometimes but not always members of the communities where they serve.

In Table 5 below, we propose 5 categories:

**Table 5:** A proposed taxonomy.

Formally employed, fully integrated within government PHC	Community members not formally employed, but actively engaged with government CB-PHC			
	a) Regular service delivery (RSD-CHW)	b) Intermediate (between a & c)	c) Episodic, occasional or time-limited service delivery	d) Community action or governance role
Pre-service training duration typically from 2–3 months up to ~2 academic years.	Training duration: from a few days to a few weeks	Training: a few days to a few weeks	Training variable, typically a few days	Training variable, typically a few days
ISCO associate professional, including auxiliary-level workers with “nurse” or “midwife” in their job title whose tasks closely align with those specified under ISCO-08: 3253. Usually full-time.	Tasks may correspond to ISCO-3253 designation Typically **≥** 10 hours/ week most of the year but generally not full-time Remunerated in some way	Tasks may correspond to ISCO-3253 designation Typically at least a few hours a week but usually < 10 Typically receive some monetary incentives	May provide services only at certain times of the year (e.g., for special campaigns), or on certain days of the month, but typically less than weekly May receive some monetary incentives	Little or no service delivery role on behalf of government PHC, & generally no remuneration
Aid Post Orderlies (*Papua New Guinea*) Auxiliary Nurse-Midwives (*India, Nepal*) Anganwadi Workers^i^ (*India*) Auxiliary Health Workers (*Nepal*) Public Health Midwives (*Sri Lanka*) Health Assistants, Family Welfare Assistants & Community Healthcare Providers (*Bangladesh*) Lady Health Workers & Vaccinators (*Pakistan*) Behvarz/ Moraghebe-Salamat (*Iran*) Health Extension Workers (*Ethiopia*) Nursing Assistants (*Uganda*) Auxiliary Nurses (Rwanda) Enrolled Nurses & Enrolled Midwives (*Uganda, DR Congo, Kenya*, & other countries in Africa) [Community Health Extension Workers (*Nigeria*)^ii^] Health Surveillance Assistants (*Malawi*) Community Health Workers (*Tanzania*) Community Health Assistants (*Zambia, Kenya*) Maternal-Child Health Aides (*Sierra Leone*) Agentes Comunitários de Saúde (*Brazil*) Agentes Polivalentes Elementares (*Mozambique*) Community Health Officers (Ghana) Agents Santé Communautaire/ Matrones (*Niger, Mali, Sénégal, Burkina Faso*) Agents Itinérants de Santé (*Burkina Faso*)	Accredited Social Health Activists (*India*) Community Health Workers (*Sierra Leone, South Africa*) Community Health Assistants (*Liberia*) Barangay Health Workers (*Philippines*) Shasthya Shebikas (*Bangladesh*)^iii^	Kaders (*Indonesia*) Female Community Health Volunteers (*Nepal*) Polyvalent CHWs, formerly Binômes & Animatrices Santé Maternelle (*Rwanda*) Village Health Workers (*Zimbabwe*) Relais Communautaires (*DR Congo*) Community-Directed Distributors & Community Health Influencers & Promoters Services (CHIPS) agents (*Nigeria*)	Village Health Volunteers (*Thailand, Papua New Guinea*) Relais (in various francophone African countries)^iv^ Volunteer CHWs (*Tanzania*) Agents Communautaires (*Madagascar*) Village Health Team members (*Uganda*)^iv^ Community Health Volunteers^iv^ (*Ghana*) Community Health Promoters (*Kenya*) Community-Directed Interventions Agents^v^ (*Nigeria* & elsewhere in Africa) TB Champions (*India*)	Members of: Village Health Committees (*India, Malawi, Namibia*) Community Health Committees (*Kenya, Mozambique*) Health Facility Committees (*Nepal, Kenya, Nigeria, Zambia*) Care Groups (*Malawi, Mozambique, Zimbabwe & other countries*)^iv^ Women’s Self-Help Groups (*India* & elsewhere in S Asia) Health Mothers Groups (*Nepal*) Local Red Cross/ Red Crescent volunteer groups (in many countries)^vi^

^i^Could be in category RSD-CHW (a). Considered “honorary workers”, given a monthly honorarium, with funds from national & state governments, considered equivalent to a salary.

^ii^Could also be categorized at “professional level”, as “paramedical practitioners” (ISCO-08:2240), given their scope of practice & 3-year duration of pre-service training.

^iii^Among the cadres listed, this one differs from the others in that it is a large-scale NGO program. For their regular duties, they could be categorized as RSD CHWs, however they are less often engaged with government PHC; their governance oversight & support are from an NGO. Their role does however entail episodic support for government campaign outreach activities.

^iv^Some may be more appropriately assigned to the intermediate category (b), particularly in localities where there is active partner support.

^v^These community members are typically mobilized mainly for special campaigns, e.g., for ivermectin distribution, as part of efforts to control onchocerciasis [24].

^vi^Members may periodically be mobilized in a time-limited or episodic service role (category c), e.g., in response to natural disasters. In some countries there are members in roles corresponding to category b.

(1) formally-employed CHWs (or auxiliary workers; they may have designations such as auxiliary nurse-midwife, but their tasks generally align relatively well with ISCO-08:3253; this category corresponds fairly closely to what Olaniran [7] designates “L2 paraprofessionals”);

and 4 categories not formally employed, classified by level of integration within government PHC:

(2) regular service-delivery CHWs, normally spending 10+ hours a week on CHW-related tasks (corresponds fairly closely to “L1 paraprofessionals” in Olaniran’s taxonomy),
(3) an intermediate category, engaged in a service delivery role, typically spending several hours a week, but fewer than 10,
(4) episodic/ occasional service delivery involvement, but no CHW-related activity most weeks, and
(5) governance or community action role, e.g., as members of a local health committee.

The last 3 categories roughly correspond to what Olaniran [7] has labeled “lay health workers”.

Note that the table below does not include *professional* health workers (what WHO refers to as “mid-level workers”) involved in CB-PHC, such as professional public health nurses or clinical officers (at the far left in Fig 1).

Programs with community members actively engaged with government CB-PHC are arrayed along a fluid continuum. In a common scenario, programs may have predominantly episodically/ occasionally-involved CHWs in districts without donor-funded partners but predominantly intermediate-level involvement (i.e., many CHWs involved most weeks) in partner-supported districts. This is a common phenomenon and points to complexities in the relationships between host government and external partners, raising such questions as: Who “owns” these programs? Who is making the decisions? [23]

Furthermore, programs change over time; where we have located specific CHW programs across this spectrum may not, in every case, reflect the current situation. As we have stated earlier, this taxonomy and the criteria used to differentiate categories are intended to be descriptive, not normative.

## Other dimensions

### Place(s) of work

Some formally-employed CHW cadres spend virtually all their time doing outreach or home visits. However, it is not uncommon that they spend at least part of their time working from a health post, dispensary or other such structure below health center level. In some programs, formally-employed CHWs spend part of every month at the health center (e.g., *Health Surveillance Assistants* in Malawi). *Health Extension Workers* in Ethiopia and *Agentes Comunitários de Saúde* in Brazil divide their time between health posts and community outreach sites (or home visits).

RSD-CHWs may visit their health post fairly frequently, for meetings for example, but they exercise their role primarily at outreach sites and in the homes of their fellow community members or, for some activities, in their own homes, e.g., for sick child care or to dispense medications or contraceptives.

### Scope of work

**Fig 2:**
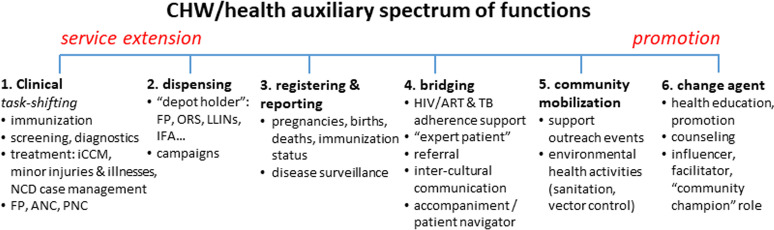
Spectrum of CHW functions. In this figure, moving from left to right: “depot holders” are community members having supplies of certain basic health commodities available to dispense to community members; “campaigns” refers to periodic mass distribution events, e.g., twice-yearly Child Health Days, during which commodities such as vitamin A, deworming medication, LLINs may be distributed and vaccines may be administered. Note that these specific activities are only illustrative; this is not meant to be an exhaustive list. NCD, non-communicable diseases (notably hypertension); FP, family planning; ANC, antenatal care; PNC, postnatal care; ORS, oral rehydration solution; LLINs, long-lasting insecticide-impregnated nets; IFA, iron-folate supplementation; ART, antiretroviral therapy.

Community-based PHC services provided by CHWs vary considerably across programs. Fig 2 presents a spectrum of functions often seen, ranging from *service extension*, such as clinical tasks and dispensing of health commodities, to *promotion* activities, such as advertising upcoming campaigns or providing educational materials, and attempting to influence care-seeking and other health-related practices in their communities [25]. The work of formally-employed CHWs typically focuses more on the service extension end of this spectrum. Regular service-delivery CHWs (not formally employed) can be involved in activities across the spectrum, although in most programs they are less involved in what could be characterized as “clinical functions” than are formally-employed CHWs (as illustrated in Fig 2, below). An important exception is sick child care, which has been introduced as part of the scope of work of RSD-CHWs in several dozen countries, under the rubric of Integrated Community Case Management (iCCM). Although they are an important component of the CHW role in many programs, such task-shifted, clinical functions (i.e., individual preventive and curative patient care) are not prominent in the ISCO-08 task description for CHWs, as outlined in Table 4. We propose that this should be addressed in subsequent revisions of ISCO.

### Beyond general government CB-PHC

In this discussion, we are focusing on general, public-sector PHC services below the level of health center, primarily in rural areas. In urban areas, commonly physicians, nurses and other health professionals are more widely available and the government provides PHC services using different delivery modalities than in rural areas, relying less on task-shifting, although CHWs may still play an important bridging role, particularly in community mobilization and patient navigation (functions towards the right end of the spectrum in Fig 2). Furthermore, service delivery is commonly more pluralistic in urban areas, with private providers playing a larger role than in rural areas, particularly in curative care. Currently there are relatively few urban CHW programs operating at scale, though we can anticipate more such programs in the future. A promising model is the Brazilian *Agentes Comunitários de Saúde* Program, with more than 380,000 agents serving across the country, mainly in urban areas.

In many settings, in addition to general CB-PHC, there are disease-specific programs engaging paid community-level staff or non-formally-employed CHWs, analogous to what is described above, e.g., in HIV and TB programs or, on a time-limited basis, in such activities as epidemic response or polio eradication. In some settings (e.g., in Pakistan), specialized cadres of community-level workers are deployed as vaccinators.

UN agencies, NGOs, non-profit and for-profit private sector institutions, and public-private partnerships may also be prominently engaged at community level and supporting CHWs, including RSD-CHWs, working on programs with looser or tighter stewardship oversight from government. Their tasks may be closely coordinated with government PHC, e.g., peer-support CHWs working in HIV programs or episodic assistance provided to government PHC counterparts for campaign-related community mobilization. It is certainly appropriate for governments, as they plan and manage services at community level, to take into account this broad range of community actors.

## Discussion/Conclusions

Health systems around the world have different histories, face different challenges, have elaborated different solutions, and are continually evolving. Nevertheless, if we are trying to generalize and learn from program experience in other settings, using more standardized terminology can help us avoid misinterpreting what is reported. In that spirit, we are proposing to the global PHC community a framework and terminology for talking about those providing services in government primary health care at community level.

There is a relatively clean distinction that can be made between those who are formally employed and working full-time and those who are neither. Among formally employed workers in CB-PHC, some can be classified—using ISCO-08 criteria—as *professional*. They would include professional nurses and clinical officers (typically with 3 or more years of preservice training, as indicated in ISCO-08). Although workers in this category are found at the most peripheral level of government PHC in urban settings in LMICs, they are less common in rural areas. The remaining formally-employed health workers in rural CB-PHC can generally be categorized by ISCO-08 criteria as *associate professional*. Many of these have tasks that align fairly closely with the ISCO-08 “community health worker” designation (3253), as outlined in Table 4. A subset of this group have “nurse” or “midwife” in their job-titles. We are proposing that, for purposes of between-country comparisons and lesson learning, it is clearer to group such occupational groups together with others having job titles more traditionally recognized as “community health workers”, focusing on actual tasks rather than formal designations.

We are also proposing not to restrict the CHW designation to those with only a few weeks or months of pre-service training (as would be the case with some earlier definitions) but also, consistent with ISCO-08 and WHO, to include workers with 1 to 2 years of pre-service training.

We have chosen not to use the term “volunteer” to designate those not formally employed. This group includes a subset who are engaged as what we are characterizing as “regular service delivery” CHWs. Our use of a cutoff of 10 hours or more per week typically involved in CB-PHC-related activities as a threshold for classifying this group as RSD-CHWs may appear somewhat arbitrary. However, this cut-off has allowed us to sort a number of programs, cleanly, between the “regular” and “intermediate” categories (e.g., India’s *ASHAs* typically exceed this threshold whereas most of Nepal’s *FCHVs* and Rwanda’s *Binômes* normally do not). As we have noted, currently RSD-CHWs—as we have defined this category—are not formally employed although generally they are remunerated in some way and in some cases their remuneration may approach the level of a low-level salaried worker (e.g., ASHAs in some parts of India). It is also worth noting that in essentially all LMIC economies, only a minority of the working-age population is engaged in formal-sector salaried employment [26].

Further to the right on our spectrum of CHWs (Fig 1 and Table 5), we have those engaged in “episodic or occasional” service, defined—again somewhat arbitrarily—as playing such a role less frequently than every week, through most of the year. In between, we have an intermediate group. An important reason for making these distinctions is that expectations are likely to vary considerably across these groups, for example with regard to remuneration. Although, in most settings, it may be unreasonable (and unfair) to expect someone to consistently put in 10 hours or more of work every week largely on an uncompensated basis, such arrangements may be judged, in the local context, to be acceptable and fair when the time commitment is substantially lower (than 10 hours/week) and the involved community members have more discretion with regard to when they’re engaged in such activities, especially if they derive other valued non-monetary benefits from their role and are supported in other ways by their communities.

It is important to recognize that the term “professional”, in addition to the ISCO designation, carries a colloquial meaning of someone engaged in skilled work for compensation (e.g., a professional athlete). This colloquial understanding is particularly relevant in the context of CHWs, many of whom have organized and demanded recognition as professionals, seeking salaries and acknowledgment of their expertise [27]. Indeed, multiple ministries of health have expressed a commitment to “professional CHWs”, in this sense, including accreditation and fair compensation—as advocated in the Monrovia Call to Action [28]. There remain significant challenges: governments having expressed a strong commitment to professionalize CHWs still have to count the cost per worker multiplied by the scale of the program and consider anticipated client reach and financial viability. Furthermore, even community health programs staffed by formally-employed CHWs often also have recourse to Women’s Clubs, Papa-Champions, Women Leaders, Youth Peer Educators, and miscellaneous Community Leaders serving a complementary function as CHWs, of a kind, without an expectation of formalized salaried employment. Some such programs would fall in the category we are proposing of “primarily engaged in governance or community action”, at the right end of the spectrum in Fig 1. Such community agents play an important role and we are not suggesting that they should be formalized.

Unlike certain schemes that have been proposed [8,9], we have not used duration of pre-service training as a primary criterion for classifying programs and, indeed, ISCO-08 gives priority attention to *tasks* rather than *training duration* in its categorization of occupational groups. In the programs we have looked at that engage formally-employed CHWs and other workers with equivalent tasks, training duration ranges from as little as a few weeks, for *Health Assistants* and *Family Welfare Assistants* in Bangladesh, up to 2 academic years (indeed, up to 3 academic years, if we include *Community Health Extension Workers* in Nigeria; however, as we have pointed out, *CHEWs* may fit better at the professional level, under ISCO-08: 2240 “paramedical practitioners”, along with such as cadres as *Clinical Officers*). Work currently underway at WHO, developing guidance on CHW training, should direct attention to important considerations in developing CHW training programs based on the skills required for their tasks, including issues such as needed duration of training.

There are implications arising from our proposal that will be *relevant to ILO* for the revision of the ISCO classification, currently underway. The task description for CHWs, under ISCO-08, captures most of the functions outlined in Fig 2, but it does not reflect the “task-shifted” clinical tasks that are common for formally-employed CHWs working in CB-PHC in LMICs (and, indeed, also for many RSD-CHWs), e.g., family planning, treatment of childhood illnesses, antenatal care, or administering vaccines. As we have suggested, the task description for CHWs (3253) should be revised accordingly in the next edition of ISCO. Furthermore, if ISCO codes 3221 (nursing associate professional) and 3222 (midwifery associate professional) are to be applied for cadres working at community level (i.e., rather than hospital or primary healthcare center), task descriptions should reflect their more autonomous roles—in contrast to their roles when working in higher-level health facilities where they are more directly supervised by health professionals.

There are also *implications for governments*. Although we are not proposing that countries change the formal designation of occupational groups, such as enrolled nurses, who are providing CB-PHC services, we do encourage governments to consider such cadres together with other categories of CHWs in their PHC human resource planning and management (indeed, they should also include professional-level workers at this level). Within the logic of the scheme we are proposing, they are grouped together with “formally employed (associate-professional) CHWs”.

As we have pointed out, many large-scale government programs falling in our category of RSD-CHWs have functions aligning well with the task descriptions for ISCO-08:3253. For such programs, governments and their development partners may want to consider whether further formalization of these CHWs would be appropriate (and feasible), including improved remuneration, supervisory support, supply of program commodities, career trajectories, and integration within health sector human resource planning and management (including related data systems). Such review and reflection may also be appropriate for programs working with cadres we are classifying as “intermediate”.

Also, we should not assume that all these issues are necessarily adequately addressed for formally-employed CHWs. For them to be effective and satisfied in their work, they also need fair (and reliable) remuneration, appropriate training, good supervisory support, reliable supply of program commodities, and attractive opportunities for career progression—dimensions of “professionalization”, as this word has been used in recent CHW discussion and debate, in the Monrovia Call to Action [28], and in WHO guidance [5]. Given the larger magnitude of potential lives saved investing in community-based PHC—vs. at facility-based PHC and hospital levels [29]— it is certainly appropriate that governments commit adequate resources and give serious attention to sound, context-appropriate planning for work at this level. Partners can play a supportive role, helping transition away from partner-driven to fully government-led, context-appropriate, community-based PHC.

Although the primary focus of this paper has been on CHWs who are integrated, at least to some degree, within government PHC services, NGOs working at community level also work with CHWs of various kinds and may also find the proposed taxonomy useful.

Health systems continue to evolve, driven by multiple forces including changing disease burdens, urbanization, professionalization of the workforce, and new technological solutions for service delivery. Nevertheless, we are confident that community-level service delivery and actors playing a boundary-spanning or bridging role between health services and the community will remain important for universal access and equity, and for individual and population health. Furthermore, it will be important to ensure dignified, decent and humanized work for the community health workforce. To build on many decades of accumulated program learning, extending back to Alma-Ata in 1978 [18] and earlier, and to draw on and apply lessons from current programs, we encourage the use of a standardized terminology, allowing us to compare apples with apples, and oranges with oranges.

**Key recommendations**

- For drawing potentially generalizable lessons from specific Community Health Worker (CHW) programs and applying them in policy and planning (including for program monitoring, evaluation, and formal reporting of national CB-PHC program activities), we recommend that ministries of health and their partners:
  o consider the tasks performed by a particular cadre, rather than their formal designation (some auxiliary-level workers not normally considered CHWs (e.g., enrolled nurses), if their duties closely align with those we associate with CHWs, should be considered together with CHWs in program planning);
  o recognize that community members engaged in some way with government primary health care (PHC) who are not formally employed span a continuum, with those at one end having important shared characteristics with formally-employed CHWs and those at the other end—who may have little or no service delivery role on behalf of government PHC—having a relationship with government PHC services that differs substantially from an employee-employer relationship.
- In its revision of the International Standard Classification of Occupations, currently underway, the International Labour Organization should add language to the task description for CHWs, reflecting the clinical tasks (curative and clinical preventive) included in the work of many CHWs in low- and middle-income countries.
- For cadres we have labeled “regular service delivery CHWs, not formally employed”, governments and their development partners should give serious consideration to whether formalization of employment would be appropriate, along with improvements in remuneration, supervisory support, supply of program commodities, career trajectories available, and integration within health sector human resource planning and management.
