# Correction: Comparing apples with apples: A proposed taxonomy for “Community Health Workers” and other front-line health workers for international comparisons

**DOI:** 10.1371/journal.pgph.0005560

**Published:** 2025-12-01

**Authors:** The PLOS Global Public Health Staff

## Notice of Republication

This article was republished on November 15, 2025, to correct errors in the affiliations that were introduced during the typesetting process. The publisher apologizes for the errors. Please download this article again to view the correct version.
